# Inhibition of mTOR improves malnutrition induced hepatic metabolic dysfunction

**DOI:** 10.1038/s41598-022-24428-7

**Published:** 2022-11-19

**Authors:** Matilda E. Arvidsson Kvissberg, Guanlan Hu, Lijun Chi, Celine Bourdon, Cino Ling, YueYing ChenMi, Kyla Germain, Ivo P. van Peppel, Linnea Weise, Ling Zhang, Valeria Di Giovanni, Nathan Swain, Johan W. Jonker, Peter Kim, Robert Bandsma

**Affiliations:** 1 Translational Medicine Program, Hospital for Sick Children, Toronto, Canada; 2 Department of Pediatrics, Section of Molecular Metabolism and Nutrition, University Medical Center Groningen, Groningen, The Netherlands; 3 The Childhood Acute Illness and Nutrition Network, Nairobi, Kenya; 4 Department of Nutritional Sciences, Faculty of Medicine, University of Toronto, Toronto, Canada; 5 Department of Biochemistry, Faculty of Medicine, University of Toronto, Toronto, Canada; 6 Cell Biology Program, Hospital for Sick Children, Toronto, Canada; 7 Centre for Global Child Health, Hospital for Sick Children, Toronto, Canada

**Keywords:** Preclinical research, Nutrition, Paediatric research, Autophagy

## Abstract

Severe malnutrition accounts for half-a-million deaths annually in children under the age of five. Despite improved WHO guidelines, inpatient mortality remains high and is associated with metabolic dysfunction. Previous studies suggest a correlation between hepatic metabolic dysfunction and impaired autophagy. We aimed to determine the role of mTORC1 inhibition in a murine model of malnutrition-induced hepatic dysfunction. Wild type weanling C57/B6 mice were fed a 18 or 1% protein diet for two weeks. A third low-protein group received daily rapamycin injections, an mTORC1 inhibitor. Hepatic metabolic function was assessed by histology, immunofluorescence, gene expression, metabolomics and protein levels. Low protein-fed mice manifested characteristics of severe malnutrition, including weight loss, hypoalbuminemia, hypoglycemia, hepatic steatosis and cholestasis. Low protein-fed mice had fewer mitochondria and showed signs of impaired mitochondrial function. Rapamycin prevented hepatic steatosis, restored ATP levels and fasted plasma glucose levels compared to untreated mice. This correlated with increased content of LC3-II, and decreased content mitochondrial damage marker, PINK1. We demonstrate that hepatic steatosis and disturbed mitochondrial function in a murine model of severe malnutrition can be partially prevented through inhibition of mTORC1. These findings suggest that stimulation of autophagy could be a novel approach to improve metabolic function in severely malnourished children.

## Introduction

Malnutrition is a global burden which is linked to 45% of all deaths in children under the age of 5^1^. The most severe form of malnutrition accounts for half a million deaths per year^1^. Despite following WHO treatment guidelines, mortality rates in acutely ill hospitalized children with severe malnutrition are still reported to be up to 20–40%^2,3^. While, these guidelines are comprised of recommendations in management of malnutrition associated complications, they are based on limited scientific evidence^4^ and understanding of the underlying pathophysiological pathways associated with severe malnutrition^5^.

Children with severe malnutrition have signs of disturbed hepatic metabolism. This manifests as hypoalbuminemia, hepatic steatosis, hypoglycemia related to decreased hepatic glucose production, and disturbed bile acid homeostasis^5–7^. The pathophysiology behind hepatic metabolic disturbances and hepatic steatosis, specifically, are not well understood. Hepatic lipid secretion appears to be preserved in malnourished children^8,9^, while lipolysis is increased and whole body lipid oxidation is decreased^10,11^. Together, increased lipolysis and decreased fat oxidation could work in tandem to enhance the flux of free fatty acids into the liver and preventing their oxidation, resulting in hepatic steatosis. This is further supported in both pre-clinical models of malnutrition and severely malnourished children that suggests that hepatic mitochondrial β-oxidation of fatty acids is impaired^6,12–14^.

Autophagy is a cellular recycling process that breaks down intracellular components in response to different stressors, such as nutrient deprivation^15,16^. It is also the major mechanism by which superfluous and damaged organelles are degraded and recycled^15^. Mammalian or mechanistic target of rapamycin complex 1 (mTORC1) is a key regulator of autophagy and cellular metabolism, and it maintains the balance between an anabolic and catabolic state in response to energy balance, nutrient availability, and growth signaling^17^. During nutrient deprivation, including amino acid starvation, mTORC1 is inhibited promoting a catabolic cellular state, which upregulates autophagy resulting in the systematically recycling of specific organelles^18–21^. This reutilization of cellular constituents helps maintain energy homeostasis and provide essential components for cell survival^22^. We previously reported in a rat model of severe malnutrition, signs of impaired hepatic autophagic flux^12^. mTORC1 inhibition has also shown to protect again hepatic steatosis in NAFLD models^23^. In the current study, we aimed to further assess the role of autophagy in a mouse model of severe malnutrition and determine whether inhibition of mTORC1 can be used as a strategy to improve hepatic metabolic dysfunction.

## Results

### Rapamycin does not affect body characteristics in low protein fed mice

**Figure 1:**
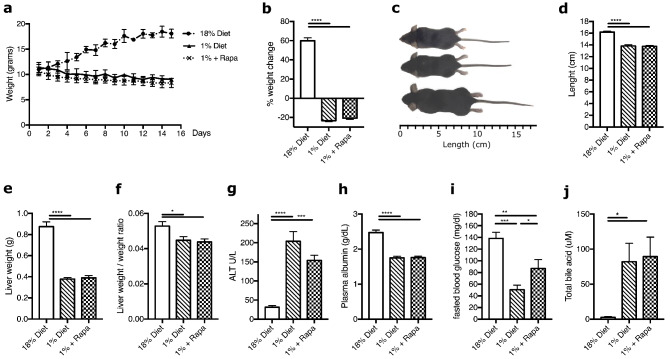
Low protein fed mice have altered morphometrics and liver function markers, with only fasting glucose levels improving by rapamycin. (**a**) Weight curve over 14 days, (**b**) Cumulative weigh change from weaning to sacrifice, %. (**c**) Photo of mice, from bottom 18% diet, 1% diet, 1% diet + Rapa. (**d**) Length at sacrifice, cm. (**e**) Liver weight (g). (**f**) Liver weight/body weight ratio. (**g**) ALT, U/L. (**h**) Plasma albumin, g/dl. (**i**) 15 h fasted glucose, mg/dl. (**j**) Total bile acids, uM. n = 6. *p < 0.05, **p < 0.005, ***p < 0.0005, ****p < 0.0001.

Wild type mice were fed either semi-synthetic control diet composed of 18% w/w protein, or an isocaloric low protein diet consisting of only 1% protein w/w (Supplementary Table S1). To examine the role of mTORC1 inhibition on the low protein diet, a separate group of 1% protein-diet mice was given daily injections of rapamycin from the first day of the diet intervention. The low protein diet induced a gradual loss of ~ 20% of their initial bodyweight (Fig. 1a,b) and resulted in a significantly smaller body length (Fig. 1c,d). When corrected for body weight, the two low protein diet groups had a significantly higher dietary intake compared to the control group throughout the two weeks, but the two low protein groups did not differ from each other (data not shown). Rapamycin treatment did not affect bodyweight change (−21.5 ± 3.5% without vs. −21.1 ± 4.8% with rapamycin treatment, *p* = 0.917, Fig. 1b) or body length (13.38 ± 0.14 cm without vs. 13.58 ± 0.22 cm with rapamycin treatment, *p* = 0.401, Fig. 1d) on a low protein diet. In accordance, liver weight was decreased in low protein compared to control diet fed mice (0.38 ± 0.01 g vs. 0.84 ± 0.03 g, *p* < 0.0001, Fig. 1e). Liver-to-body weight ratio was also reduced in low protein-fed mice (0.045 ± 0.002 vs. 0.051 ± 0.001 ratio, *p* = 0.016, Fig. 1f) compared to controls. Both absolute and relative liver weight were not affected by rapamycin treatment (Fig. 1e,f).

### Rapamycin partially prevents low protein diet-induced hepatic dysfunction

The low protein diet-fed mice showed signs of liver injury and decreased function. To assess liver damage, we measured plasma ALT, which was increased by the low protein diet compared to the control diet (203.8 ± 25.4 U/L vs. 31.4 ± 4.4 U/L, *p* < 0.0001, Fig. 1g) and considered abnormal (reference ALT plasma levels are 25–80 U/L in mice^24^), reflecting liver cell injury in mice on the low protein diet. Rapamycin treatment did not significantly decrease plasma ALT levels (153.8 ± 13.7 U/L with rapamycin, *p* = 0.129, Fig. 1g). Low plasma albumin (hypoalbuminemia), a protein produced and secreted by the liver, is a sign of severe malnutrition and is indicative of liver synthetic function^25^. Plasma albumin was reduced in the low protein mice compared to controls (1.75 ± 0.05 g/dL vs. 2.48 ± 0.07 g/dL, *p* < 0.0001, Fig. 1h), reflecting impaired liver synthetic function, but was not affected by rapamycin treatment (1.76 ± 0.05 g/dL, *p* = 0.950, Fig. 1h). The ability of the liver to maintain blood glucose levels during fasting is crucial for survival. Fasting blood glucose levels were reduced in low protein fed mice compared to controls (50.4 ± 7.9 mg/dl vs. 138.4 ± 10.4 mg/dl, *p* = 0.0001, Fig. 1i) and this reduction was partially prevented by rapamycin (86.9 ± 15.3 mg/dl vs. 50.4 ± 7.9 mg/dl, *p* = 0.049, Fig. 1i).

We have previously reported that malnourished children have increased plasma levels of bile acids, consistent with cholestasis^7^. The increased bile acid concentrations could be due to impaired liver function and/or be contributing to a decline in liver function^26,27^. Total plasma bile acid concentration in low protein-fed mice were increased 82.1 ± 26.4 uM vs. 2.7 ± 1.0 uM in the control diet (*p* = 0.037, Fig. 1j), mostly driven by increases in cholic acid (CA), taurocholic acid (T-CA), β-muricholic acid (β-MCA), and tauro-muricholic acid α & β (TMCA-α&β) (Supplementary Fig. S1A). Normal serum bile acid concentrations in mice range between 1 and 20 uM^28^, indicating cholestasis in our model. Rapamycin treatment on the low protein diet did not affect the total concentration of plasma bile acids (Fig. 1j), but lowered levels of some conjugated bile acids such as glycocholic acid (GCA), TMCA-α & β, and taurochenodeoxycholic acid (TCDCA) as seen in Supplementary Fig. S1A. The low protein diet significantly decreased expression of genes central to bile acid synthesis, i.e., Cytochrome P450 family 7 subfamily A member 1 (*Cyp7a1)* and Cytochrome P450 family 27 subfamily A member 1 (*Cyp27a1)* (Supplementary Fig. S1B). *Cyp7a1* but not *Cyp27a1* expression was restored to control diet levels by rapamycin treatment (Supplementary Fig. S1B). The low protein diet reduced gene expression of the sodium-taurocholate co-transporting polypeptide (NTCP), which is important for bile acid uptake from the blood into hepatocytes, and the expression of NTCP was also restored by rapamycin (Supplementary Fig. S1B). Gene expression of the bile salt export pump (BSEP), responsible for secreting bile acids into the bile canaliculi, was unchanged in the low protein fed mice and unaffected by rapamycin treatment (Supplementary Fig. S1B).

Altogether, these results indicate that a low protein diet leads to changes in hepatic metabolism, which can be, in part, improved by rapamycin treatment.

### Rapamycin improves hepatic steatosis in low protein-fed mice

**Figure 2:**
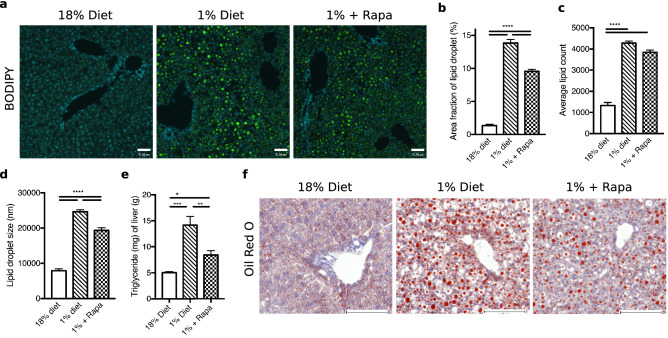
Mice on a low protein diet develop hepatic steatosis, which is partially mitigated by rapamycin. (**a**) BODIPY staining, scale bar 49um. (**b**) Area fraction of lipid droplets in percent. (**c**) Average lipid count. (**d**) Average lipid droplet size, nm. (**e**) Triglyceride quantification, mg/g, n = 6. (**f)** Oil Red O staining, scale bar 100 um. *p < 0.05, **p < 0.005, ***p < 0.0005, ****p < 0.0001.

Steatosis is one of the hepatic hallmarks observed in severely malnourished children. Hepatocytes from low protein-fed mice developed large inclusions of neutral lipids as shown by BODIPY staining (Fig. 2a). Quantitative analysis of BODIPY staining confirmed that lipid content in hepatocytes of low protein-fed mice was increased (Fig. 2b,c) and this was associated with an increase in average lipid droplet size (Fig. 2d). Rapamycin treatment reduced hepatic lipid droplet content (Fig. 2a,b), associated with a reduction in the size of lipid droplets (Fig. 2a,d). In accordance with these results, hepatic triglyceride content was also increased in low protein diet-fed mice compared to controls (14.2 ± 1.7 mg/g vs. 5.0 ± 0.2 mg/g, *p* = 0.0001) with partial improvement after rapamycin treatment (8.4 ± 0.8 mg/g, *p* = 0.005, Fig. 2e). These results were also consistent with lipid staining using oil red O (Fig. 2f) and observed morphology of large hepatic vacuoles seen with H&E staining of hepatocytes from low protein fed mice, most prominently seen in the periportal and intermediary zones (Supplementary Fig. S2). These results demonstrate that hepatic triglyceride content is increased in low protein diet-fed mice and is that this can be prevented by rapamycin treatment.

### Rapamycin partially prevents mitochondrial damage observed in low protein fed mice

**Figure 3:**
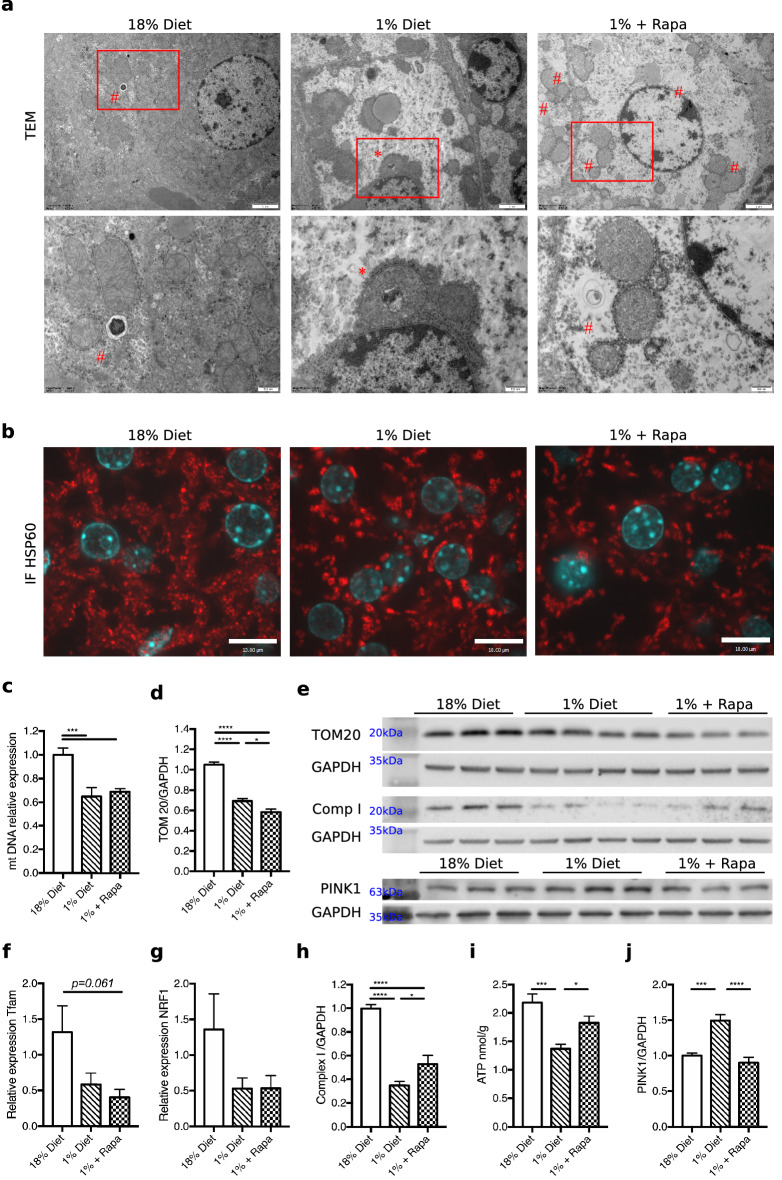
Low protein fed mice illustrate changed mitochondrial morphology and markers of function which were mitigated by rapamycin. (**a**) TEM magnification × 4000, scale bar 2um, red square encircles a mitochondria with an inclusion body, # indicate autophagosomes. (**b**) HSP60 IF staining in red, scale bar 10um. (**c**) mitochondrial DNA. (**d**) TOM20 quantification of western blot relative to 18% diet. (**e**) Westernblots. (**f**) Relative expression Tfam. (**g**) Relative expression NRF1. (**h**) Complex I quantification of western blot relative to 18% diet. (**i**) Hepatic ATP levels, nmol/g wet weight. (**j**) PINK1 quantification of western blot relative to 18% diet. The westerns were cut prior to incubation with the primary antibodies, the full size blots can be seen in Supplementary Fig. S7. *p < 0.05, **p < 0.005, ***p < 0.0005, ****p < 0.0001.

Ultrastructural analysis revealed that the morphology of mitochondria was altered in hepatocytes of low protein diet-fed mice. TEM analysis showed that mitochondria in the low protein-fed mice were elongated and enlarged, with some having inclusion bodies (Fig. 3a, inclusion body indicated by *). The mitochondria of low protein-fed mice treated with rapamycin were also enlarged but had fewer inclusion bodies (0.013 ± 0.004 inclusion body per mitochondria) compared to the mice on the low protein untreated groups (0.042 ± 0.001, *p* = 0.018, Fig. 3a). The TEM quantification of mitochondria showed a loss of mitochondria number under the low protein condition. The average mitochondria per cell under the control diet was 109.3 ± 7.0, compared to 21.9 ± 1.0 in the untreated low-protein group (*p* < 0.0001) and 22.5 ± 1.1 in the rapamycin group (*p* < 0.0001 compared to control). The low protein-diet induced alterations in mitochondrial elongation were further illustrated by immunofluorescence staining of heat shock protein 60 (HSP60), an intra-mitochondrial marker (Fig. 3b). The HSP60 staining appeared to also show a visual reduction in the number of mitochondria in the low protein-fed mice. The low protein-diet induced changes in mitochondria number were supported by a reduction in relative mtDNA expression (*p* = 0.008, Fig. 3c) and TOM20 protein expression (*p* < 0.0001, Fig. 3d,e), a mitochondrial outer membrane marker, compared to control mice. Interestingly, low protein diet-fed mice treated with rapamycin displayed the lowest expression of TOM20 (*p* = 0.025) while their mtDNA levels were similar to untreated low protein diet-fed mice (Fig. 3c–e). The loss of mitochondrial membrane and maintained mitochondrial DNA levels and numbers may suggest increased turnover of enlarged mitochondria specifically in the rapamycin group, although ultrastructural analyses do not clearly confirm this. We were unable to detect clear evidence for a decrease in mitochondrial biogenesis in our model, although there was a trend towards a decrease. This was illustrated by mitochondria biogenesis markers Tfam (control diet vs. low protein *p* = 0.123, vs. rapamycin group *p* = 0.061, Fig. 3f) and NRF1 (control diet vs. low protein *p* = 0.200, vs. rapamycin group *p* = 0.232, Fig. 3g).

We next assessed changes in markers of mitochondrial function in our model. We found that low protein-fed mice had decreased protein expression of mitochondrial electron transport chain complex I (*p* < 0.0001, Fig. 3h and Supplementary Fig. S3) which was improved by rapamycin treatment (*p* = 0.019, Fig. 3h and Supplementary Fig. S3). We found that the low protein diet had decreased expression of mitochondrial electron transport chain complex IV (*p* = 0.046, Supplementary Fig. S4), however, complex IV protein expression was not improved by rapamycin treatment (*p* = 0.571 low protein vs. rapamycin, Supplementary Fig. S4). The effect of low protein diet feeding on electron transport was associated with lower ATP content compared to controls (1.37 ± 0.08 nmol/g vs. 2.19 ± 0.15 nmol/g, *p* = 0.0007, Fig. 3i) which was also improved by rapamycin treatment (1.83 ± 0.12 nmol/g, *p* = 0.043, and *p* = 0.121 rapamycin to controls Fig. 3i). We next determined the levels of mitochondrial damage marker PINK1^29^, to see if an increase in damaged mitochondria could explain the impairment in cellular respiration, illustrated by loss of ATP and complex I expression. We found that PINK1 was higher in low protein-fed mice indicating an increase in damaged mitochondria (*p* = 0.0003, Fig. 3j and Supplementary Fig. S3). This level was restored to control levels with rapamycin treatment suggesting a decrease in damaged mitochondria (*p* < 0.0001, Fig. 3j and Supplementary Fig. S3).

**Figure 4:**
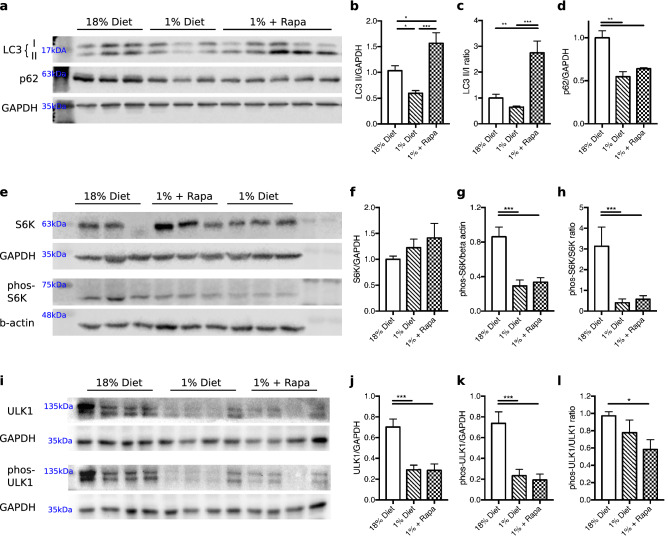
Rapamycin treatment significantly increased autophagy markers. (**a**). Western blots LC3, p62, GAPDH. Western blot quantification of and shown as a relative expression to 18% control diet (**b**) total LC3-II/GAPDH. (**c**) LC3-II to I ratio. (**d**) p62/GAPDH. (**e**) Western blots S6K, GAPDH, phos-S6K, beta-actin. Western blot quantification of and shown as a relative expression to 18% control diet (**f**) S6K/GAPDH. (**g**) phos-S6K. (**h**) phos-S6K/S6K ratio. (**i**) Western blots ULK1, GAPDH, phos-ULK1 (**j**) ULK1/GAPDH. (**k**) phos-ULK1/GAPDH. (**l**) phos-ULK1:ULK ratio. The westerns were cut prior to incubation with the primary antibodies, the full size blots can be seen in Supplementary Fig. S9. *p < 0.05, **p < 0.005, ***p < 0.0005, ****p < 0.0001.

Since PINK1 levels accumulate on damaged mitochondria to signal mitophagy^29^, we examined two markers of autophagy: p62, an autophagy adaptor protein and LC3 protein, an autophagy effector protein that is cleaved to form LC3-II on formation of autophagosomes^30^. The low protein diet led to a reduction in total LC3-II protein fraction compared to the control diet (*p* < 0.036, Fig. 4a,b and Supplementary Fig. S5 for full blots), but not the LC3-II/I ratio (*p* = 0.391, Fig. 4a,c and Supplementary Fig. S5). The lower level of LC3-II could indicate a reduction in autophagosome formation in low protein diet mice compared to controls. However, TEM analysis showed a similar average of autophagosomes per cell in the low protein diet group as in the control group (1.09 ± 0.17 in the untreated low protein group and 1.13 ± 1.17 in the control group, *p* = 0.856). However as autophagy is a dynamic flux, additional flux experiments are needed to substantiate these findings. Rapamycin treatment markedly increased LC3-II protein fraction in low protein diet-fed mice compared to both a normal control diet controls and untreated low protein diet-fed mice (*p* = 0.256 and *p* = 0.004 respectively, Fig. 4a,b and Supplementary Fig. S5) suggesting that autophagy was stimulated in rapamycin treated animals. This was further supported by rapamycin’s upregulation of LC3-II/I ratio compared to the control and the untreated low protein diet (*p* = 0.001 and *p* = 0.0003 respectively, Fig. 4a,c and Supplementary Fig. S5) and by an increased number of autophagosomes per cell compared to untreated low protein diet-fed mice as assessed by TEM (2.76 ± 0.33 autophagosome per cell vs. 1.09 ± 0.17, *p* < 0.0001).

Protein levels of p62 were decreased by low protein diet feeding (*p* = 0.0002, Fig. 4d and Supplementary Fig. S5) but its levels were unaffected by rapamycin treatment. The low protein diet inhibited mTORC1 activation as shown by decreased phosphorylated S6K versus the control diet (*p* = 0.0007, Fig. 4e,h and Supplementary Fig. S5), this downregulation was not further decreased by rapamycin (low protein vs. rapamycin *p* = 0.905, Fig. 4e,h and Supplementary Fig. S5). To further examine autophagy regulation in low protein diet animals, we next examined the autophagy initiating factor ULK1, which is inhibited by active mTORC1 through the phosphorylation of ULK at Ser757^31^. Interestingly, we observed a reduction in the ULK1 protein level in the low protein diet liver, which was not improved by rapamycin (*p* = 0.997, Fig. 4i–k and Supplementary Fig. S5). We found that the Ser757 phosphorylation ratio was not significant different between the low protein diet versus normal diet animals (*p* = 0.420, Fig. 4i–l and Supplementary Fig. S5). However, the ULK1 phosphorylation ratio of the rapamycin treated animals was significantly lower compared to the normal diet animals (*p* = 0.043, Fig. 4i–l and Supplementary Fig. S5). These combined results suggest that during prolonged period of amino acid starvation, there is mTOR inhibition but potentially does not lead to an upregulation of autophagosomes. However, we caution against overinterpreting phosphorylated: total ULK1 ratio given the strong effect on ULK1 content in the low protein-diet groups, which can be due to autophagy-mediated degradation or decreased protein synthesis as seen under prolonged amino acid deficiency^32^. Short treatment of rapamycin has shown to upregulate AKT phosphorylation^33^, in our model we saw no effect on AKT phosphorylation by the low protein diet or the rapamycin administration compared to the control group (*p* = 0.741 and *p* = 0.725 respectively, Supplementary Fig. S6). The suggested increase in autophagy seen in the rapamycin treated mice may explain the reduction in TOM20 and the recovery of PINK1 to control levels. However, as autophagy is an active flux additional experiments are needed with an autophagic inhibitor, to determine if autophagy is truly upregulated by rapamycin.

Together, these data indicate that low protein feeding in young mice leads to disturbances in mitochondrial function which can be improved by rapamycin treatment, potentially by clearing dysfunctional mitochondria through increased autophagy.

### Rapamycin prevents disturbances in central carbon metabolism in mice fed a low protein diet

**Figure 5:**
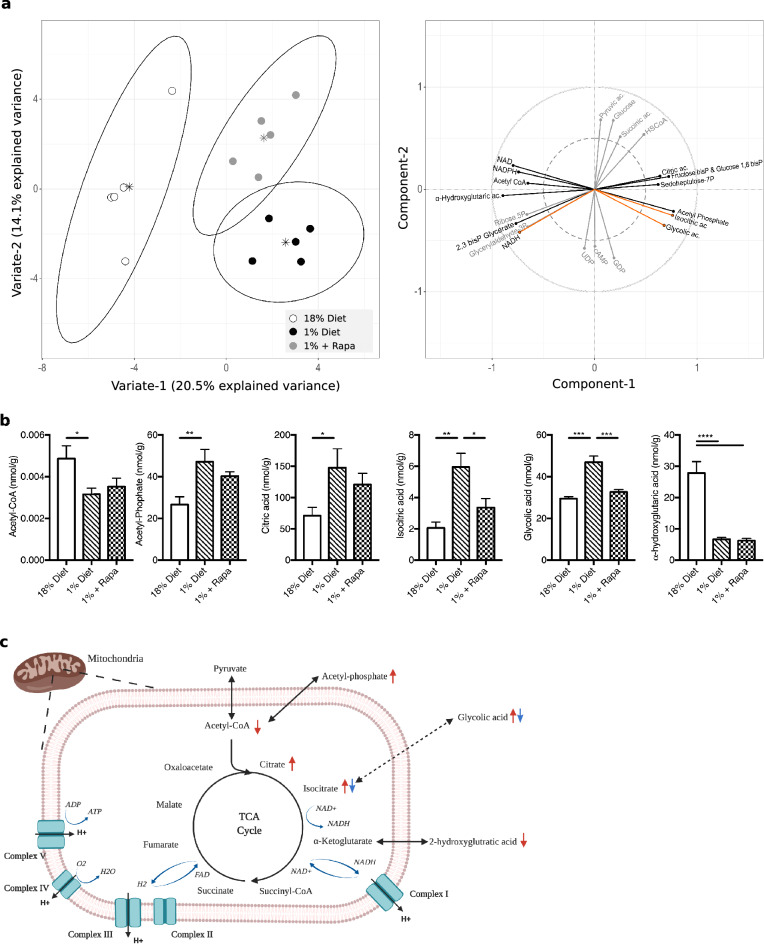
A low protein diet impairs the TCA cycle, which is in part prevented by rapamycin treatment. (**a**) Left panel, score plot of PLS-DA showing group separation based on all measured metabolites (groups coded as per legend); Right panel, correlation plot presenting the relationships between the top distinctive metabolites (VIP > 1); the metabolites that significantly contribute to component-1 (i.e., control vs. low protein fed) are indicated with black lines, those in grey show metabolites that only contribute to component-2 (i.e., difference between rapamycin treated vs. untreated low protein fed animals); while metabolites that contribute to both component-1 and -2 are indicated by orange lines. (**b**) Bar plots presenting mean and SEM of selected top metabolites that most discriminate between groups, nmol/g. n = 5. (**c**) Schematic overview of the changes in metabolites seen in the low protein fed-mice, red arrow indicating the increase or decrease in metabolites compared to control mice, and blue arrow compared to rapamycin. *p < 0.05, **p < 0.005, ***p < 0.0005, ****p < 0.0001.

Our findings of hepatic steatosis in combination with reduced ATP content suggested impairments in energy homeostasis which could also be modulated by rapamycin treatment. To investigate this, we used targeted metabolomics to measure, in liver, the metabolites involved in the central carbon metabolism. Using PLS-DA together with univariate analysis, we demonstrated that rapamycin treated animals had distinctive metabolic profiles compared to either controls or low protein fed animals (Fig. 5a: left panel, showing group separation; right panel, correlation plot of top discriminative variables). The main differentiating features were that, compared to controls, the low protein fed mice had lower levels of acetyl-CoA and of several NAD factors while showing increased levels of citric and isocitric acid together with increased acetyl-phosphate (Fig. 5a,b, Supplemental Table S2 and Supplementary Fig. S7). This pattern is consistent with impairments in the tricarboxylic acid (TCA) cycle as depicted in Fig. 5c. This impairment did not lead to an increase in lactic acid indicating that anaerobic glycolysis was not upregulated (Supplementary Fig. S8, which also includes remaining TCA metabolites). Certain metabolite changes were prevented, at least in part, by rapamycin treatment, with treated animals showing lower levels of isocitric acid and glycolic acid. However, rapamycin treatment also tended to heighten levels of pyruvic acid, glucose and succinic acid while further lowering NADH (Fig. 5a, and Supplementary Fig. S7–9). Together, these data suggest that low protein diet feeding impairs the TCA cycle with likely consequences on hepatic ATP production and mitochondrial function and that rapamycin treatment can potentially prevent certain metabolites from accumulating, aid in redirecting intermediates out of the liver, or induce alternative pathways.

## Discussion

Metabolic dysregulation is associated with an increased risk of mortality in children with severe malnutrition. Our findings demonstrate that 2 weeks of low protein diet feeding in weanling mice induce phenotypic and metabolic changes that mimic those observed in early childhood severe malnutrition. We further show that low protein feeding in weaning mice is associated with: (1) disturbances in mitochondrial homeostasis and function, (2) insufficient capacity to maintain cellular energetics, and (3) accumulation of damaged mitochondria. Using this model, we demonstrate that pharmacological inhibition of mTORC1 using rapamycin improved hepatic mitochondrial and partially metabolic function.

Early studies in the 1960s and 70 s used either protein or caloric restriction as pre-clinical models of severe malnutrition^12,34–49^. These models exhibited body weight loss, sometimes stunting and varying degrees of hepatic steatosis. This variability in symptomatology is attributable to different levels of protein restriction, length of the dietary intervention, age and type of animal used (often rats). We previously reported on hepatic metabolic dysfunction a rat model of malnutrition. However, this model is less amenable to genetic manipulation and is more costly than a mouse model. Our mouse model of severe malnutrition described in this study developed signs of wasting, stunting, and hepatic steatosis, and with additional important clinical features described in children with severe malnutrition^5,50–52^ including signs of hepatic dysfunction such as hypoalbuminemia, hypoglycemia and cholestasis^5,7^. In addition, our model presented with signs of disturbed hepatic mitochondrial homeostasis and function, which has been reported in our rat model of severe malnutrition and severely malnourished children^12–14,53,54^.

Our results suggest that an inability to effectively degrade damaged mitochondria contributes to the impaired mitochondrial function observed in the low protein-fed mice. PINK1 is a mitophagy activator that is rapidly degraded in healthy mitochondria but accumulates on damaged mitochondria to signal mitophagy by recruiting Parkin^55,56^. Although, the accumulation of PINK1 suggest that damaged mitochondria were marked for degradation, low LC3-II levels and lack of an increase in autophagosome content could suggest an insufficient autophagosome formation, preventing optimal autophagy in mice chronically fed a low protein diet. However this would require directly supportive data, such as autophagy flux assays and assessment of specific mitophagy markers such as Parkin and Drp1 in isolated mitochondria. However, mTORC1 inhibition with rapamycin induced an upregulation of both LC3-II protein levels and autophagic structures in electron micrographs, consistent with increased autophagosome formation. These data together with the observed decreased PINK1 levels, suggest that rapamycin may promote autophagy and a reduction in dysfunctional mitochondria.The improved hepatic levels of Complex I, ATP levels and TCA cycle intermediates further support an overall improved mitochondrial function in rapamycin-treated animals. However, this requires direct supportive data, for example by measuring mitochondrial respiration.

Our murine model demonstrated increased total plasma bile acids, consistent with observations described in severely malnourished children^7^. Limited data from pre-clinical models of malnutrition and malnourished children suggest that the increased levels in bile acids lead to FXR activation and an increase in FGF-19 subsequently leading to suppressed hepatic bile acid synthesis, illustrated by decreased *Cyp7a1* expression^57^. In the current study we found a similar decrease in hepatic *Cyp7a1* expression, as well as in *CYP27a1* expression (Supplementary Fig. S2B). The increased bile acid levels seen in our model are therefore most likely associated with an increase in FXR activation. FXR activation has shown to transcriptionally decrease NTCP expression through increased SHP expression and interaction with retinoic acid receptor (RAR). This leads to reduced hepatocyte bile acid uptake and protects against cytotoxic hepatic bile acid accumulation^58^. FXR activation is also known to suppresses autophagy in an mTOR independent manner^59,60^, and could thus contribute to the potentially lack of increased autophagosome formation that was seen in the low protein fed mice.

Our study has several limitations. A limitation of our model is that we did not restrict caloric intake with malnutrition being induced by feed a low protein diet. Malnutrition in children is generally thought to occur as a consequence of having a diet that is low in both calories and protein. Different animal models have been used historically to induce malnutrition that included either feeding a low protein diet or dietary restriction. However, food restriction was not found to be ethical by our animal ethics committee and we therefore could not implement a model that included food restriction. Severely malnourished children often present with acute and chronic infections, such as gastroenteritis and HIV. Our current model did not allowed us to study the metabolic changes induced by a low protein diet in the context of an infection. Additionally, as an mTORC1 inhibitor, rapamycin can trigger diverse metabolic effects beyond inducing autophagy and these vary depending on acute or chronic administration. mTORC1 is a nutrient sensor that promotes anabolic respiration and cell proliferation regulating both glucose and lipid metabolism^22,61^. In NAFLD studies, for example, rapamycin has shown to alleviate steatosis by downregulating lipid metabolic enzymes and hepatic lipid uptake receptors^62,63^ and in this study we did not address these other metabolic effects of rapamycin.

In conclusion, inhibiting mTORC1 prevented malnutrition-induced hepatic metabolic dysfunction likely via improved mitochondrial function. This study highlights a new potential area for therapeutic interventions to metabolically stabilize children with severe malnutrition particularly those admitted to hospital who have a high risk of early mortality.

## Methods

### Animal model

Wild type C57BL/6 J (The Jackson Laboratory, Bar Harbor, USA) mice were maintained at the Laboratory Animal Services facility at the Hospital for Sick Children, Toronto, Canada. Male 21-day old weanling mice were randomly assigned to either a semi-synthetic control (18% protein, Tekland Laboratories, USA) or an isocaloric low protein (1% protein, Tekland Laboratories, USA) diet for two weeks. The protein in the low protein diet was replaced by an isocaloric amount of starch while other nutritional components were kept identical. Diet compositions are presented in Supplementary Table S1. Food and water were provided *ad libitium*. Intraperitoneal injection of rapamycin (6 mg/kg/day) was started at weaning, concomitantly with the 1% diet intervention. Rapamycin was diluted first in 100% ethanol to make 50 mg/ml stock solution, and then further in 10% Tween80 (P1754, Sigma, USA). All animal procedures met the requirements of the Ontario Animals for Research Act (R.S.O. 1990, c.A.22) and the federal Canadian Council on Animal Care, as required by the Hospital for Sick Children animal ethics committee. The Animal Use Protocol (AUP) was approved by animal care committee (ACC) ethical review board at Lab Animal Services (LAS) of the Peter Gilgan Centre for Research and Learning (PGCRL), Toronto. All methods were carried out in accordance with relevant guidelines and regulations. The study was carried out in compliance with the ARRIVE guidelines.

### Histology and immunofluorescence

Liver histology analysis was performed on tissues selected from the hepatic left lateral lobe with one sample fixed overnight in 4% paraformaldehyde (PFA) or washed in 1 × Phosphate-buffered saline (PBS) then cryoprotected through serial dehydration with sucrose for embedding and freezing in optimal cutting temperature (OCT, Sakura Fintek, Torrance, CA, USA) compound. Frozen tissue was cut at 10 μm and then stained with Oil Red O solution or boron-dipyrromethene 493/503 (BODIPY) (D3922, Invitrogen, Eugene, OR, USA). PFA fixed tissue were wax-embedded and cut at 4 μm and stained with hematoxylin and eosin (H&E) or immunofluorescent labels. For this, antigen retrieval was performed for 3 min in a pressure cooker in Tris–EDTA buffer (pH 9.0). Sections were blocked with 30% BSA/goat serum, and incubated with primary antibodies overnight at 4 °C. Sections were incubated with either Alexa Fluor 488/594 goat anti-mouse or anti-rabbit secondary antibodies (1:5000, Thermo Fisher Scientific, Rockford, IL, USA) and 4’,6-diamidino-2-phenylindole (DAPI) (Sigma, Toronto, ON, Canada) for 1 h at room temperature, mounted in VectaShield (Vector Labs, Burlingame, CA, USA), and visualized using a confocal microscope.

### Transmission electron microscopy

Fresh liver samples were cut into 1–2 mm^2^ and fixed in 2% glutaraldehyde in 0.1 M sodium cacodylate buffer pH 7.3 at 4 °C. Tissues were subsequently fixed, rinsed, post-fixed, rinsed, dehydrated, and infiltrated as follows: 10 min in 0.1 M sodium cacodylate buffer with 0.2 M sucrose (pH 7.3), 1.5 h 1% osmium tetroxide in 0.1 M sodium cacodylate buffer (pH 7.3), 10 min in 0.1 M sodium cacodylate buffer with 0.2 M sucrose (pH 7.3), 10 min in 70% ethanol, 10 min in 90% ethanol, 3-times 10 min in 100% ethanol, 2-times 10 min in propylene oxide, 1 h in 50/50 propylene oxide/Quetol-Spurr resin, 1 h in Quetol-Spurr resin and overnight in Quetol-Spurr resin. Sections (90 nm) were cut on a Leica Ultracut ultramicrotome (Lecia Microsystem, Concord, ON, Canada), stained with uranyl acetate and lead citrate and imaged by TEM viewed in a FEI Tecnai 20 transmission electron microscope (FEIon Company, Hillsboro, OR, USA). Quantification of autophagosomes and mitochondria (with and without inclusion bodies) was done single blinded by selecting 15 cells per samples with magnification at 3000 × to image the whole cell and sections of the cells at 5000 × for the quantification.

### Immunoblotting

Liver samples (30 mg) were homogenized in 300ul volume of ice-cold lysis buffer (Invitrogen) with 15ul of protease inhibitor (Invitrogen). Liver lysate was centrifuged at 15,000 g for 15 min at 4 °C. Protein concentration was measured using the BCA protein assay kit as per manufacturer’s instructions (Thermo Fisher Scientific, Mississauga, ON, Canada). Protein (20 mg) was resolved on a 4–12% gradient gel (Invitrogen) and transferred to a PVDF membrane using the Blot System (Invitrogen, Burlington, ON, Canada). After transfer, membranes were blocked at room temperature in 5% BSA made in TBS with 0.1% Tween-20 for 1 h. The membranes were cut prior to incubation, to carry out multiple western blots per membrane. Membranes were incubated at 4 °C overnight with primary antibody, followed by secondary antibody incubation for 1 h at room temperature. An overview of the antibodies can be found in Supplementary Table S3. Membranes were visualized using Pierce enhanced chemiluminescence.

### Biochemical analysis

To determine triglyceride (TG) content, lipids were extracted from approximately 50 mg of frozen liver homogenized in 20 volumes of 2:1 chloroform–methanol mixture and incubated at 4 °C for a 48 h. Samples were washed with 0.2 volumes of 0.9% NaCl, the upper phase was suctioned off and the chloroform and methanol left to evaporate over several days. Remaining lipid was dissolved in 100% ethanol and TG were quantified using biochemical kits (Randox Laboratories, London, UK). Photometric absorbances were read at 500 nm. TG values were normalized to protein concentrations from the same liver tissue as measured with a BCA kit (Thermo Fischer Scientific). Adenosine triphosphate (ATP) levels in liver was determined using the ATP Colorimetric/Fluorometric Assay kit (Abcam, Toronto, ON, Canada; n = 6 mice per group). Measurements were performed following manufacturer's instructions on 10 mg of frozen tissue after perchloric acid and potassium hydroxide deproteinization. ATP was measured in duplicate and expressed as µmol per gram of wet tissue. Plasma alanine transaminase (ALT) levels were measured using an activity assay kit (Abcam), and plasma albumin was measured using a colometric assay kit (Abcam).

### DNA-based quantification of mitochondria

To calculate the mitochondrial DNA (mtDNA) to genomic DNA ratio, total DNA was isolated from 10 mg of liver samples in 300ul of TRIzol (Invitrogen). Samples were homogenized with a metal bead at 30 Hz for 1.5 min. Then centrifuged at 15,000 rpm for 10 min, and the supernatant was collected and vortexed with 300ul of 95–100% ethanol. DNA was purified using the Direct-Zol (Zymo research, Burlington, ON, Canada), and the DNA concentration measured using NanoDrop (Thermo Fisher Scientific). mtDNA primers (forward: 5’-CCC AGC TAC TAC CAT CAT TCA AGT-3’; reverse: 5’-GAT GGT TTG GGA GAT TGG TTG ATG T-3’). 500 ng of genomic DNA were used for each qPCR reaction, with Advanced qPCR Mastermix with Supergreen Dye (Wisent Bioproducts, St-Bruno, Canada). qPCR was performed using a CFX384 Touch Real-Time PCR Detection System (Bio-Rad, Mississauga, Canada).

### qPCR

cDNA was synthesized from 500 ng of liver total RNA using the SuperScript VILO cDNA Synthesis Kit (Thermo Fisher Scientific). For qPCR, 0.1ul of cDNA were used with Advanced qPCR Mastermix with SuperGreen Dye (Wisent Bioproducts). For measurement of mtDNA number, 500 ng of genomic DNA were used for each qPCR reaction. qPCR was performed using a CFX384 Touch Real-Time PCR Detection System (Bio-Rad). Ribosomal protein L13a (Rpl13a) was used as the housekeeping gene.

### Bile acid analysis

Plasma bile acids were quantified at the Sick Kids Analytical Facility for Molecules by liquid chromatography mass spectrometry (LC–MS) using the Biocrates Life Sciences Bile Acids Kit (Biocrates, Innsbruck, Austria) and analyzed with the Agilent UHPLC 1290 LC system coupled to an ABSciex QTRAP 5500 in negative ESI MRM mode. The samples were lyophilized, reconstituted with 75% ethanol and homogenized. Then, vortexed for 5 min followed by a 10 min centrifugation at 20,000 g, the supernatants were used for bile acid measurement following manufacturer’s instructions.

### Central carbon metabolism

This analysis was carried out at the Metabolomics Innovation Center in Edmonton, Canada. Frozen liver samples were weighed and homogenized in 2 uL/mg water followed by addition of 8 μL/mg methanol. Homogenization was carried out for 1 min twice at 30 Hz with two 4 mm metal balls on a MM 400mill mixer. The sample was then sonicated for 3 min and centrifuged at 15,000 rpm for 20 min at 5ºC. Supernatants were collected and analyzed by ultra-performance liquid chromatography—tandem mass spectrometry (UPLC-MS) (Dionex 3400, Thermo Fischer Scientific) as previously described for: (1) glucose and selected sugar phosphate^64^; (2) other phosphatases containing metabolites and nucleotides, and (3) TCA cycle carboxylic acid^65^. A detailed description of the methods and the internal standards used can be found in Supplementary Materials S1.

### Statistical analysis

Replicate measures of morphometrics, hepatic triglycerides, ATP levels, plasma albumin, 15 h fasting glucose, ALT levels, and immunoblot densitometry were analyzed using either: (1) one-way ANOVA with a Bonferroni’s or false discovery rate (FDR) multiple comparison test for parametric data, or (2) a Kruskal–Wallis test with a Dunn’s multiple comparison test for nonparametric data (Prism version 7). All values are represented as means ± standard error of the mean with an unadjusted or a multiple comparison-adjusted *p* value < 0.05 considered significant. BODIPY image analysis was carried out using ImageJ (version 1.52), using the 18% protein wild type stainings to set the analysis threshold. Analysis of central carbon metabolism was carried out using sparse partial least-squares multivariate analyses (sPLS-DA) using the mixOmics package (R version 4.0.2). First, we conducted visual assessment for outliers in either measurements or samples using histograms and principal component analysis, and none were detected. Then, we used sPLS-DA to select and rank top discriminative features using ℓ^1^ regularization. Prior to analysis, variables were Box-Cox transformed to achieve normal distribution before standardization. Overall model performance was evaluated using average error rate < 30% based on Mahalanobis distance obtained through leave-one-out cross-validation and area under Receiver Operating Characteristic curve (AUROC) > 0.7. sPLS-DA procedures were then used to select the top 20 differentiating variables loading on either Component-1 or -2 which were tested for selection stability across folds. Final selection of variable was based on the combined criteria of showing on either Component-1 or -2: (1) VIP score > 1.0, (2) stability of selection in top 20 variables > 80% and (3) correlation > 0.5. Group differences were confirmed and further explored using linear regression, with a Gaussian distribution and identity link function, performed on Box-Cox transformed variables.

## Supplementary Information

Supplementary Information.
