# Supplementary material for: Inhibition of mTOR improves malnutrition induced hepatic metabolic dysfunction
Source: Sci Rep. 2022 Nov 19;12:19948. doi: 10.1038/s41598-022-24428-7 (PMC9675758; doi:10.1038/s41598-022-24428-7)
Supplement: Supplementary file 1 — Supplementary Information. [file 41598_2022_24428_MOESM1_ESM.pdf]

Supplementary Information

Inhibition of mTOR improves malnutrition induced hepatic metabolic dysfunction

Matilda E. Arvidsson Kvissberg[1,2], Guanlan Hu[1], Lijun Chi[1], Celine Bourdon[1,3], Cino Ling[1], YueYing ChenMi[1], Kyla Germain[5,6], Ivo P. van Peppel[2], Linnea Weise[2], Ling Zhang[1], Valeria de Giovanni[1], Nathan Swain[1,4], Johan W. Jonker[2], Peter Kim[5,6], Robert Bandsma[1]*

[1] Translational Medicine Program, Hospital for Sick Children, Toronto, Canada.

[2] Department of Pediatrics, Section of Molecular Metabolism and Nutrition, University Medical Center Groningen, Groningen, The Netherlands.

[3] The Childhood Acute Illness and Nutrition Network.

[4] Department of Nutritional Sciences, Faculty of Medicine, University of Toronto, Toronto, Canada.

[5] Department of Biochemistry, Faculty of Medicine, University of Toronto, Toronto, Canada.

[6]Cell Biology Program, Hospital for Sick Children, Toronto, Canada.

[7] Centre for Global Child Health, Hospital for Sick Children, Toronto, Canada.

*robert.bandsma@sickkids.ca

Supplementary Table S1. Diet composition

Components	18% Protein Control Diet (g/kg)	1% Protein Intervention Diet (g/kg)
Casein	207	11,5
DL-Methionine	2,7	0,36
Sucrose	350	350
Corn Starch	251,3	426,54
Maltodextrin	50	50
Corn Oil	52,6	4,2
Cellulose	41,06	60,6
Vitamin Mix#	10	10
Ethoxyquin, antioxidant	0,01	0,01
Mineral Mix*	13,37	13,37
Calcium Phosphate	17,36	22,37
Calcium Carbonate	4,6	1,1
Nutrient % kcal from		
Protein	19,6%	1,1%
Fat	13,2%	13,3%
Carbohydrate	67,2%	85,6%
Kcal/g	3,7	3,7

Supplementary Fig. S1A - Plasma bile acids

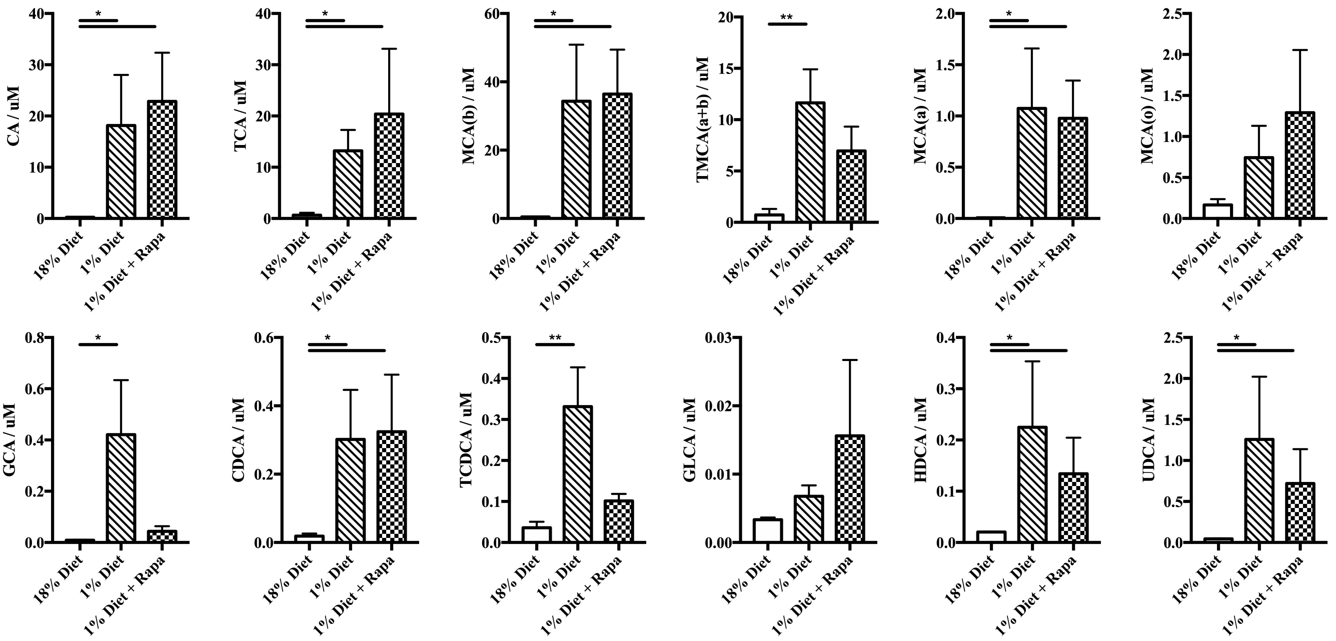

Supplementary Fig. S1B - Hepatic bile acid genes

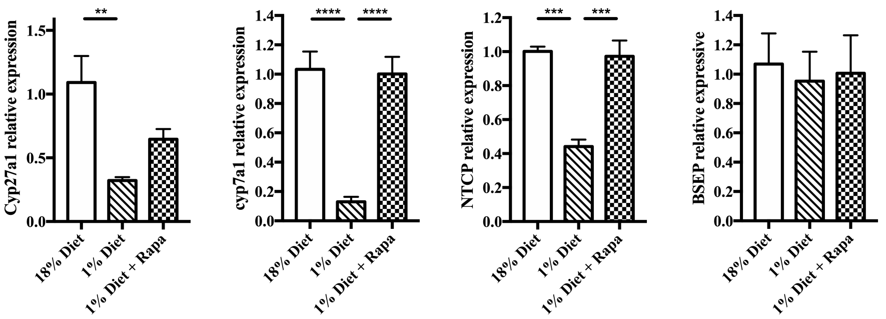

Individual bile acids in uM measured by LC-MS: cholic acid (CA), taurocholic acid (TCA), glycotauric acid (GCA), muricholic acid-beta (MCA-b), chenodeoxycholic acid (CDCA), taurochenodeoxycholic acid (TCDCA), glycolithocholic acid (GLCA), hyodeoxycholic acid (HDCA), ursodeoxycholic acid (UDCA). n=5. Relative expression of bile acid enzymes and transporters. n=6. * $p<0.05$, ** $p<0.005$, *** $p<0.0005$, **** $p<0.0001$.

Supplementary Fig. S2 - Hematoxylin and eosin staining image

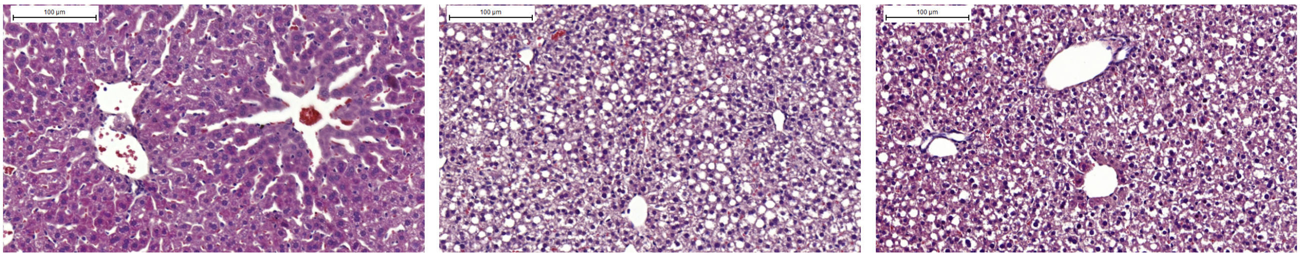

18% Protein Diet | 1% Protein Diet | 1% Diet + Rapa

Supplementary Fig. S3 - Uncut westernblots from Fig 3e

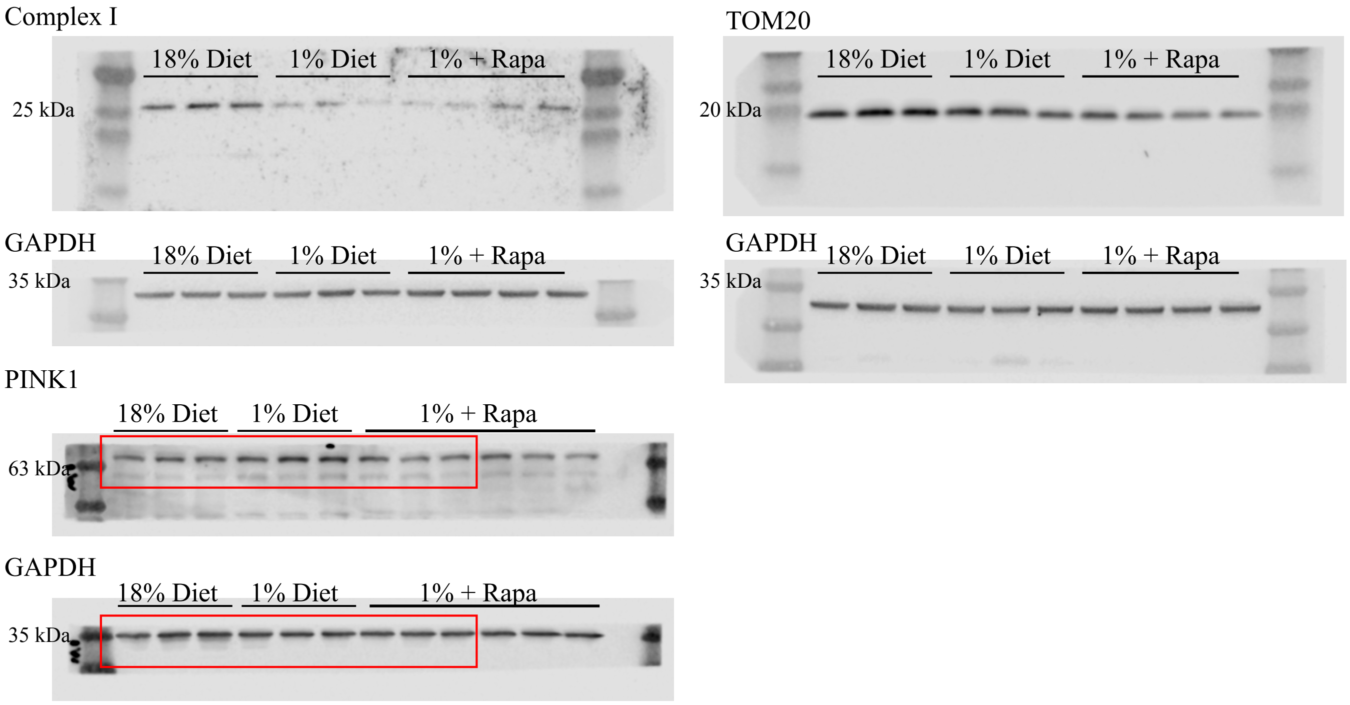

Red box indicates the westerns part of the western that is shown in Fig 3e, if not the whole blot is shown

Supplementary Fig. S4 - Westernblot Complex IV

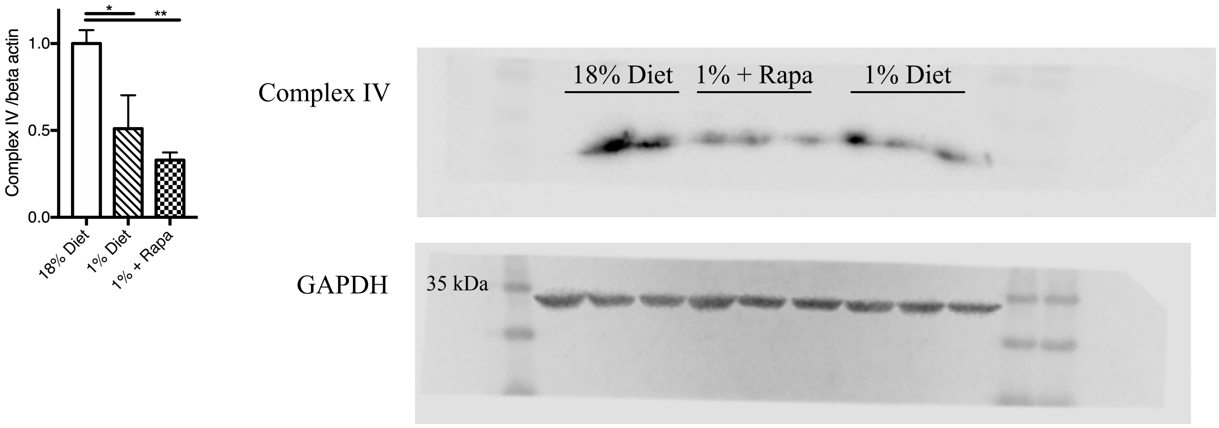

Supplementary Fig. S5 - Uncut westernblots from Fig. 4

Blots were cut before antibody incubation

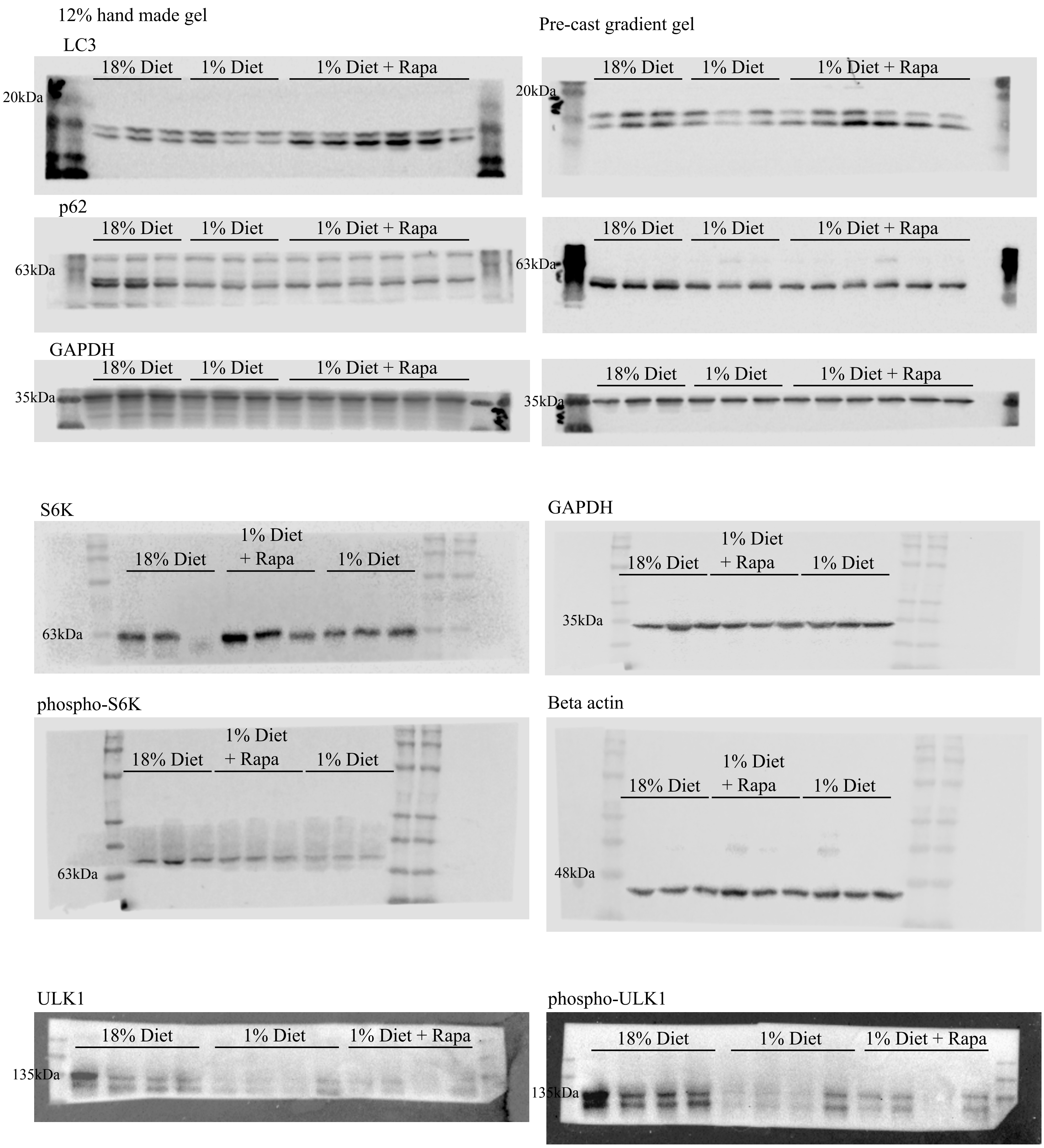

Supplementary Fig. S6 - AKT western blots

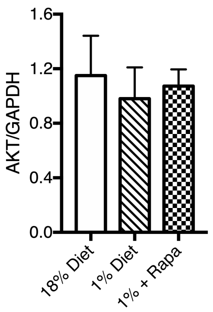

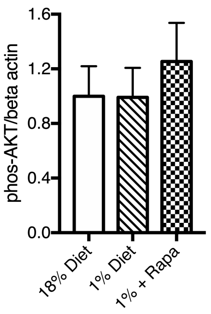

AKT

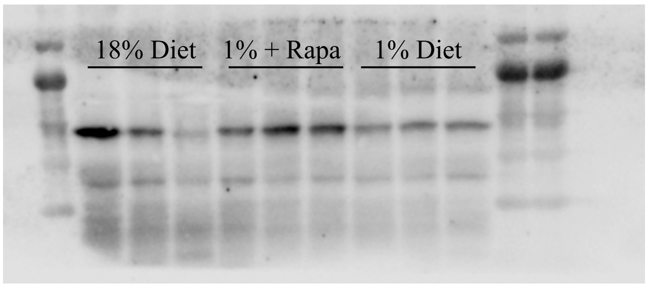

Phospho-AKT

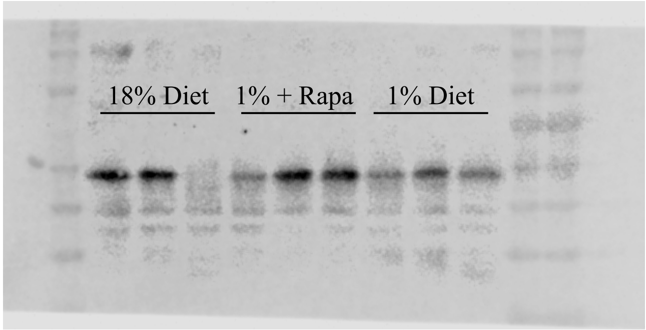

GAPDH

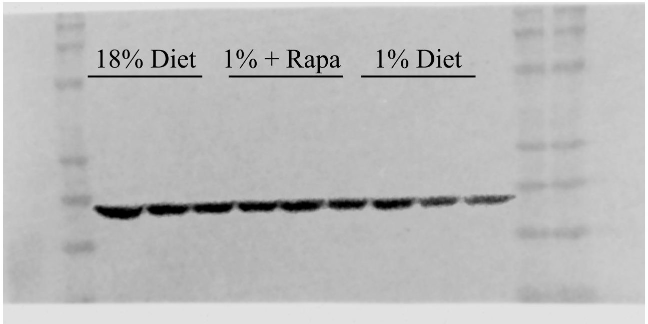

Beta actin

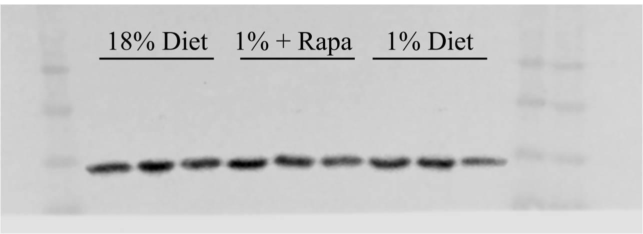

Supplementary Table S2 PLS-DA and VIP analysis

Metabolite	sPLS Comp1	Freq Comp1	sPLS VIP Comp1	sPLS Comp2	Freq comp2	sPLS VIP Comp2	Selected on	GLM Component 1 p value GLP Gc	GLM Component 2 p value GLP Gr
α-hydroxyglutaric acid	-0,42	1	3,02	0,00	0,07	2,27	C1	**<0.0001**	0,46
NAD+	-0,33	1	2,34	0,00	0,07	1,76	C1	**<0,001**	0,23
NADPH	-0,29	1	2,05	0,00	NA	1,54	C1	**0,01**	0,90
NADH	-0,25	1	1,76	-0,25	0,93	1,77	C1 & C2	**0,01**	0,09
2,3-bisP glycerate	-0,24	1	1,68	0,00	NA	1,27	C1	**0,01**	0,78
Glycerylaldehyde-3P	-0,19	1	1,35	0,00	0,07	1,02	C1	0,05	0,62
Acety-CoA	-0,18	1	1,32	0,00	NA	0,99	C1	**0,01**	0,58
Ribose-5P	-0,17	1	1,20	0,00	0,07	0,90	C1	0,06	0,84
Cyclic AMP	0,00	NA	0,00	-0,39	1,00	1,83	C2	0,38	0,07
GDP	0,00	NA	0,00	-0,32	1,00	1,48	C2	0,20	0,08
UDP	0,00	NA	0,00	-0,24	1,00	1,13	C2	0,78	0,15
Succinic acid	0,00	NA	0,00	0,31	1,00	1,44	C2	0,82	0,12
Glucose	0,00	NA	0,00	0,32	1,00	1,52	C2	0,81	0,11
Pyruvic acid	0,00	NA	0,00	0,35	1,00	1,63	C2	0,43	0,09
Hs CoA	0,11	0,87	0,77	0,20	0,93	1,09	C2	0,18	0,18
Citric acid	0,16	1	1,14	0,00	NA	0,86	C1	**0,01**	0,54
Sedoheptulose-7P	0,22	1	1,60	0,00	0,07	1,20	C1	**0,01**	0,89
Fructose-bisP + Glucose-1-6-bisP	0,22	1	1,60	0,00	NA	1,21	C1	**0,01**	0,95
Acetyl phosphate	0,27	1	1,95	0,00	0,07	1,47	C1	**<0,01**	0,31
Glycolic acid	0,29	1	2,08	-0,42	1,00	2,51	C1 & C2	**<0.0001**	**<0.0001**
Isocitric acid	0,29	1	2,08	-0,11	1	1,65	C1 & C2	**<0.0001**	**0,01**

Supplementary Fig. S7 - CCM metabolites VIP>1 from PLS-DA analysis and significant in GLM analysis Component 1-Diet

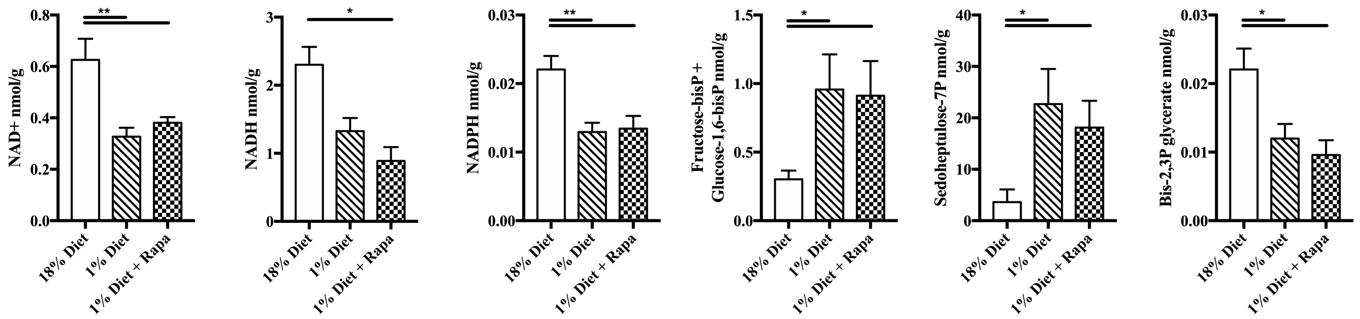

Supplementary Fig. S8 - Lactic acid and the remaining TCA metabolites not depicted in Fig. 5b

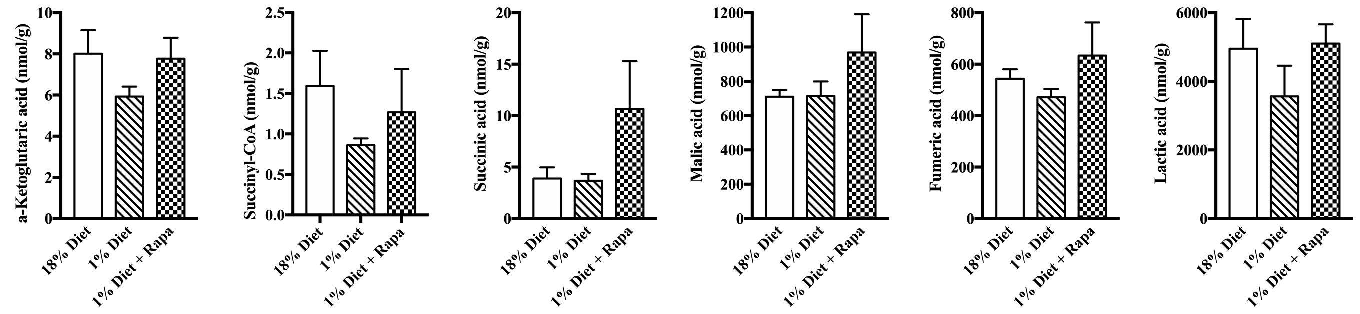

Supplementary Fig. S9 - CCM metabolites VIP>1 from PLS-DA Component 2 - Treatment

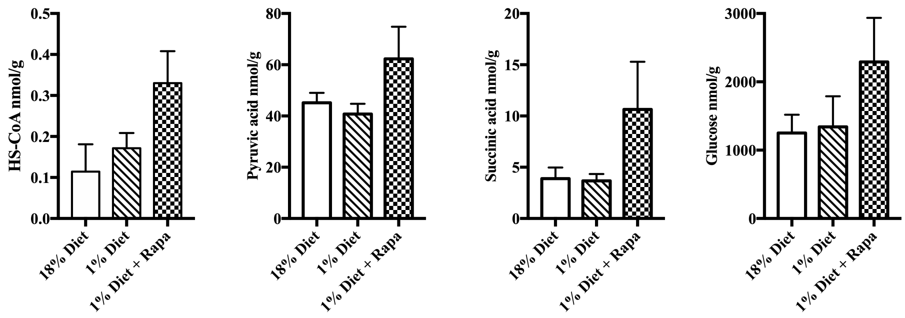

Supplementary Table S3. Antibodies overview

**Antibody**	**Brand**	**Catalog number**
Complex I (1:1000)	Abcam Biotechnology	Ab16731
Complex IV (1:1000)	Abcam Biotechnology	Ab16056
DRP1 (1:1000)	Abcam Biotechnology	Ab56788
GAPDH (1:5000)	Novus Biotechnology	NB-300-221
HSP60	Abcam Biotechnology	Ab46798
LC3B (1:1000)	Cell Signaling Technology	2775
MFN2 (1:2000)	Santa Cruz Biotechnology	sc-515647
P62 (1:1000)	Novus Biotechnology	NBP1-48320
PINK1 (1:1000)	Novus Biotechnology	BC100-494
TOM20 (1:1000)	Santa Cruz Biotechnology	Sc-11415
Phos-ULK1 (1:1000)	Cell Signaling Technology	14202
ULK1	Cell Signaling Technology	8054
DAPI	Sigma	D9564
Goat anti-rabbit Alexa Fluor 568 (1:5000)	Thermo Fischer Scientific	A-11011
Goat anti-mouse Alexa Fluor 488 (1:5000)	Thermo Fischer Scientific	A-28175

Supplementary materials S1. Quantitation of CCM metabolites in mouse liver by UPLC-MRM/MS

UPLC-MS system

A Dionex 3400 UHPLC system coupled to a 4000 QTRAP mass spectrometer was used. The MS instrument was operated in the multiple-reaction monitoring (MRM) mode with negative-ion (-) or positive-ion (+) detection, depending on which groups of metabolites were measured.

Metabolite extraction

Each liver tissue sample was weighed frozenly into an Eppendorf tube. Water, at 2 µL per mg tissue, was added and the samples were homogenized for 1 min twice at a shaking frequency of 30 Hz, with the aid of two 4-mm metal balls, on a MM 400 mill mixer. After a short-time centrifuge, methanol, at 8 µL per mg tissue, was added and the samples were homogenized again for 1 min twice using the same settings. The samples were then sonicated in an ice-water bath for 3 min, followed by centrifugal clarification at 15,000 rpm and 5 ^{o}C in an Eppendorf 5424R centrifuge for 20 min. The clear supernatants were collected for the following assays.

Quantitation of TCA cycle carboxylic acids

A standard stock solution of 10 TCA cycle carboxylic acids was prepared in 80% methanol as S1 (200

to 1000 nmol/mL each compound). This standard solution (S1) was serially diluted at 1 to 4 (v/v) with the same solvent to make standard solutions S2 to S10. 20-µL aliquots of each standard solution and the clear supernatant of each sample were mixed with 20 µL of an internal standard solution containing 9 ^{13}C- or deuterium-labeled analogues of the TCA cycle carboxylic acids (except isocitric acid), 20 µL of a 3-NPH.HCl solution and 20 µL of an EDC.HCl solution. The mixture was allowed to react at 30 ^{o}C for 30 min. After reaction, 200 µL of water was added and 10-µL aliquots of the resultant solutions were injected onto a C18 UPLC column (2.1x100 mm, 1.7 um) to quantitate the TCA cycle carboxylic acids by UPLC-MRM/MS with (-) ion detection, according to the procedure described in our publication (Han, et al., Electrophoresis. 2013; 34(19): 2891–2900).

Quantitation of glucose and selected sugar phosphates

Serially diluted standard solutions (S1 to S9) containing glucose, glucose-6P, mannose-6-P, ribose-5P, erythrose-4P and glyceralaldehyde-3P were prepared in 80% methanol. 50-µL aliquots of each of the standard solutions and the clear supernatant of each sample solutions were mixed with 50 µL of a solution containing $^{13}C_6$-glucose and $^{13}C_6$-glucose-6P as internal standards, 50 µL of 25 mM AEC solution and 100 µL of 50 mM NaCNBH3 solution and 20 µL of acetic acid. The mixtures were allowed to react at 60 ^{o}C for 70 min. After reaction, 300 µL of water and 300 µL of chloroform were added to each tube. The mixtures were vortex mixed for 30 s at 3,000 rpm, followed by centrifugation at 10,000 rpm for 10 min. The aqueous phase, in 20-µL aliquots, was injected onto a 15-cm long UPLC column to quantitate glucose, glucose-6P, mannose-6P, ribose-5P, erythrose-4P and glyceralaldehyde-3P by UPLC-MRM/MS, using the same procedure as described in our publication (Han, et al. Analytical Chemistry, 2013; 85(12): 5965–5973).

Quantitation of other phosphate-containing metabolites and nucleotides

100 µL of each supernatant was mixed with 100 µL of a $^{13}C_{10}$-labled GTP (internal standard) solution, and 200 µL of chloroform. The tubes were vortex-mixed for 30 s, followed by centrifugal clarification. The upper aqueous phase was separated, taken out and dried in a nitrogen evaporator at 30 ^{o}C under a gentle nitrogen gas flow. The residues were reconstituted in 400 µL of water. In addition, a standard mix containing targeted phosphate-containing metabolites was dissolved in a solution containing the same internal standard to prepare standard solutions of S1 to S9 at a serial dilution ratio of 1 to 4 (v/v). 10-µL aliquots of the sample solutions and the standard solutions were injected onto a polar C18 UPLC column (2.1 x 150 mm, 1.7 um) for gradient elution using a custom-developed ion-pairing LC-MRM/MS method with ammonium buffer (A) and acetonitrile (B) as the binary solvents for gradient elution. The efficient gradient was from 15% to 50% B in 12 min, at a flow rate of 250 µL/min and a column temperature of 40 ^{o}C. The metabolites were detected by UPLC-MRM/MS with (-) ion detection.

Supplementary Table S4

	Weight change %	Length at sacrifice	Liver weight	Liver: weight ratio	Plasma ALT U/L	Plasma Albumin g/dL	Plasma Fasted glucose mg/dl	Total Bile acid uM	Hepatic Triglycerides mg/g liver
	66,9	15,3	0,689	0,051	53,009	2,198	158	6,545	4,4973
	52,5	16	0,891	0,0506	19,118	2,653	141	1,576	5,4522
	47,0	16,6	0,956	0,0549	19,611	2,209	162	2,387	5,6550
	65,4	16,5	0,833	0,0484	22,737	2,593	114	1,653	5,0273
	68,0	16	0,889	0,0529	32,279	2,596	79	1,177	4,2489
18% Diet	54,3	15,5	0,807	0,0557	31,588	2,759	99		5,2258
	79,6	16	0,756	0,0429	46,099	2,434	176		
	60,8	15,7	1,195	0,0728	26,357	2,556	196		
	52,5	17	0,973	0,0532		2,277	138		
	60,3	16,8					141		
	42,5	16,4					118		
	69,0	16,5	0,754	0,0459					
	-21,1	13,2	0,318	0,0353	322,169	1,649	94	140,59	10,0144
	-24,0	14	0,306	0,0419	182,571	1,587	68	70,388	13,5828
	-23,8	13,2	0,383	0,0498	293,213	1,806	78	37,103	12,0668
	-21,2	12,9	0,388	0,0579	166,201	1,816	40	142,412	16,3600
1% Diet	-25,8	13,1	0,346	0,0443	168,011	1,907	38	77,442	11,3668
	-26,5	14,6	0,44	0,0454		1,906	21		21,5337
	-18,2	15	0,412	0,0451	100,885	1,859	34		
	-24,4	14	0,401	0,0400	186,437	1,657			
	-26,5	14,5	0,412	0,0479	211,280	1,567	43		
	-22,3	13,7	0,380	0,0403			38		
	-23,3	13,5	0,481	0,0540	143,168	1,55	98	12,818	6,5557
	-20,4	13,4	0,252	0,0341	230,530	1,657	52	19,991	6,6300
	-20,0	14,1	0,451	0,0490	126,387	1,853	139	24,17	9,8523
	-17,9	14,4	0,482	0,0473	126,716	1,726	206	115,821	11,7939
1% Diet + Rapa	-15,2	14	0,386	0,0406	154,438	1,694	144	144,491	7,4241
	-18,7	13,9	0,384	0,0441	159,620	1,684	104	142,139	8,2864
	-18,0	14	0,33	0,0402	135,682	1,753	43		
	-26,5	13,2				1,88	46		
	-17,9	13,4				2	29		
	-21,2	14,7	0,39	0,0448			57		
	-24,4	13,4	0,375	0,0441			69		
	-26,5	13,2	0,363	0,0398			56		

Supplementary Table S5

Lipid average size			Percentage lipid area			Lipid count		
18% diet	1% diet	1% + Rapa	18% diet	1% diet	1% + Rapa	18% diet	1% diet	1% + Rapa
6366	22795	19207	0,925	10,733	8,218	1839	5956	2637
5602	22994	21025	0,637	10,104	10,068	1439	5558	2951
6701	24297	19297	0,398	9,91	9,816	751	5159	3135
6674	22292	18385	0,484	10,081	9,567	917	5720	3207
7213	22734	21267	1,248	9,56	9,345	2188	5319	2708
6083	24175	18778	0,359	9,96	8,501	747	5211	2790
4940	24620	20609	0,193	8,603	8,531	495	4420	2551
4355	21614	23079	0,205	8,049	11,1	596	4710	2964
4315	26368	17410	0,145	7,828	7,842	424	3755	2776
5146	26306	20993	0,423	8,991	9,626	1040	4323	2826
12396	26061	19339	1,641	9,945	9,295	1675	4827	2962
16008	27081	13555	2,015	10,054	3,812	1592	4696	3557
10458	21607	17000	1,629	7,721	5,723	1970	4520	4258
13944	22958	17541	1,279	7,758	6,687	1160	4274	4822
21566	23147	16951	2,617	9,198	6,417	1535	5026	4788
16336	25771	16995	1,817	11,656	6,128	1407	5721	4561
14709	22226	19066	1,899	8,788	6,789	1633	5001	4504
14333	27208	17640	2,228	13,008	6,195	1966	6047	4442
9360	31865	19428	0,617	13,097	6,468	834	5199	4211
10568	25826	19081	1,047	10,168	6,511	1253	4980	4316
7262	23886	18845	2,942	8,02	7,066	2497	4247	4743
7267	21533	18468	3,058	15,024	6,408	2593	4300	4389
5286	21284	30231	1,105	13,206	11,262	1288	3824	4712
8489	22533	31275	4,587	12,373	11,725	3330	3384	4742
8279	23256	31939	4,659	12,781	11,267	3468	3387	4462
7837	20379	41438	3,861	11,957	13,006	3036	3616	3970
6754	23184	31915	2,812	13,099	11,559	2566	3482	4581
8186	25389	38847	3,616	17,019	16,864	2722	4131	5491
7402	25435	39202	3,329	18,267	14,074	2772	4426	4541
8370	25151	34188	4,578	16,467	12,125	3371	4035	4486
8558	24832	33383	4,973	14,977	12,312	3581	3717	4665
15326	23679	31794	1,027	15,818	11,337	413	4117	4510
4099	19373	12114	0,288	15,957	7,556	433	5076	3844
4559	19852	12178	0,307	15,835	8,3	415	4916	4200
12658	19150	14492	0,711	15,969	5,531	346	5139	2352
11887	19442	13871	0,447	15,585	8,587	232	4940	3815
9646	20232	13732	0,595	16,752	7,208	380	5103	3235
3793	20214	14968	0,255	15,708	6,15	414	4789	2532
3682	18529	15070	0,266	14,9	5,754	446	4956	2353

4472	19438	16163	0,2	14,979	6,447	276	4749	2458
5056	19506	14413	0,306	15,759	6,537	373	4979	2795
4816	19460	15398	0,314	16,382	6,544	402	5188	2619
4900	31075	18358	0,484	22,766	7,307	609	4515	2453
5339	33042	16148	0,632	21,156	13,216	729	3946	5044
5431	32692	16793	0,754	22,444	14,636	855	4231	5371
5283	27836	15223	0,711	19,422	12,415	829	4300	5026
5455	35706	17567	0,696	22,474	14,714	786	3879	5162
5840	33995	18036	0,858	24,155	15,517	906	4379	5302
5312	29285	15294	0,461	22,224	12,131	535	4677	4888
4849	36081	16684	0,304	23,407	12,735	387	3998	4704
5753	34798	15875	0,731	24,551	12,436		4348	4828
5143	35794	14018	0,364	24,771	11,146		4265	4900
5107	39699	14865	0,641	24,085	10,893		3739	4516
	22613	14228		11,525	9,944		3141	4307
	25848	19124		13,401	10,318		3195	3325
	23424	17689		11,501	10,19		3026	3550
	25390	13768		13,278	8,382		3223	3752
	27134	19037		14,635	11,479		3324	3716
	28583	17073		15,301	9,608		3299	3468
	25124	14715		12,03	7,196		2951	3014
	23976	14568		11,986	7,451		3081	3152
	22006	14945		13,155	8,856		3684	3652
	24728	14981		13,943	9,038		3475	3718
	23797	14874		12,534	7,644		3246	3167
	23786	15736		11,467	9,486		2971	3715
	25084	18460		14,36	11,64		3528	3886
	16885	18496		10,812	11,179		3946	3725
	23328	16290		13,457	9,465		3555	3581
	23499	19187		12,698	10,872		3330	3492
	22309	19722		10,527	10,881		2908	3400
	21572	19952		9,594	11,364			
	20697	17312		8,97	10,975			
	21025	21026		9,242	11,348			
	20970	15838		9,98	9,331			
	19949	15810		9,581	8,853			
		19171			10,934			

Supplementary Table S6

Sample Identification	Material		CA	CDCA	DCA	GCA	GCDCA	GDCA	GLCA	GLCAS	GUDCA
			Concentration [µM]								
		LLOQ	0,03	0,02	0,02	0,03	0,02	0,01	0,01	0,05	0,01
		ULOQ	75	30	10	75	20	10	5	15	10
58 - BT7 Plasma 18%	plasma	10	38,200	0,424	0,004	0,117				0,060	
59 - BT8 Plasma 18%	plasma	10	50,600	0,933	0,001	0,046		0,002	0,004	0,005	0,003
60 - BW1 Plasma 18%	plasma	10	0,454	0,036	0,414	0,003		0,004		0,003	
61 - BW2 Plasma 18%	plasma	10	0,373	0,011	0,152	0,006					0,003
62 - BW3 Plasma 18%	plasma	10	0,235	0,026	0,115			0,001		0,004	
63 - BT1 Plasma. 1%	plasma	10	55,400	0,362		1,190	0,013			0,008	0,010
64 - BT2 Plasma. 1%	plasma	10	16,900	0,051	0,001	0,465			0,004	0,008	0,003
65 - BV4 Plasma 1% (V looks like U)	plasma	10	15,200	0,842	0,002	0,026				0,002	0,004
66 - BV5 Plasma 1% (V looks like U)	plasma	10	2,860	0,119	0,002	0,024			0,002	0,009	0,005
67 - BU2 Plasma 1% (used to say BT)	plasma	10	0,434	0,136	0,004	0,399	0,014	0,000			0,008
68 - BT3 Plasma rapa	plasma	10	0,139			0,013		0,002		0,003	0,003
69 - BT4 Plasma rapa	plasma	10	0,117	0,003	0,003	0,011		0,001			
70 - BV1 Plasma rapa	plasma	10	2,770	0,077		0,008				0,004	
71 - BV2 Plasma rapa (V looks like U)	plasma	10	3,550	0,029		0,005		0,003		0,004	
72 - BV3 Plasma rapa	plasma	10	19,200	0,159	0,002	0,042				0,005	0,002

LEGEND

- Valid
- No Peak
- < LLOQ
- > ULOQ

HDCA	LCA	MCA(a)	MCA(b)	MCA(o)	TCA	TCDCA	TDCA	TLCA	TLCAS	TMCA(a+b)	TUDCA	UDCA
0,01	0,01	0,005	0,01	0,005	0,02	0,01	0,01	0,01	0,02	0,01	0,01	0,02
5	5	5	10	5	50	20	10	5	10	10	15	30
0,076	0,070	1,260	32,000	0,331	69,700	1,460		0,003	0,032		0,428	0,361
0,406	0,079	1,920	73,900	4,160	3,590	0,135	0,003	0,002		3,930	0,101	2,330
0,023	0,054	0,009	0,552	0,393	0,182	0,020	0,043			0,125	0,016	0,060
0,019		0,006	0,308	0,206	0,269	0,037	0,064		0,028	0,130	0,030	0,042
0,020		0,006	0,360	0,207	0,067	0,009	0,030			0,044	0,018	0,036
0,233		2,600	57,200	1,790	10,300	0,324	0,002		0,025	9,650	0,221	1,310
0,026		0,075	4,060	0,089	7,640	0,182	0,004	0,002	0,026	7,380	0,139	0,083
0,717		2,400	87,300	1,580	12,900	0,221	0,002		0,026	16,700	0,340	4,180
0,115		0,266	21,300	0,247	28,900	0,703	0,003	0,003	0,025		0,990	0,604
0,034		0,033	1,820	0,005	6,360	0,228	0,001	0,002		3,160	0,094	0,110
0,019		0,006	0,497	0,005	2,480	0,091	0,007	0,002	0,025	3,030	0,219	0,036
0,022		0,008	0,651	0,012	0,299	0,022	0,018		0,027	0,307	0,048	0,055
0,029		0,111	10,200	0,201	2,900	0,071	0,003			3,450	0,070	0,097
0,032		0,114	7,870	0,171	5,280	0,072	0,036	0,002	0,026	6,740	0,161	0,106
0,128		1,480	58,100	1,590	20,400	0,127	0,002	0,003	0,037	13,700	0,176	0,710

Supplementary Table S7

Western Blot	18% Diet	1% Diet	1% Diet + Rapa	qPCR	18% Diet	1% Diet	1% Diet + Rapa
TOM20/ GAPDH	0,94442064	0,7300925	0,67140632	mtDNA	0,95747	0,508652	0,654885
	1,03461852	0,7359426	0,61173848		1,11468	0,668987	0,637652
	1,02096083	0,54265807	0,54160505		0,92784	0,769697	0,752659
	1,03700125	0,65071071	0,64771204		0,94891	0,719342	0,711772
	1,14819616	0,77568164	0,63355979		1,07650	0,639175	
	1,1591118	0,64261194	0,41097518				
	1,03461852	0,7300925	0,61173848				
	1,02096083	0,7359426	0,54160505				
Complex I/ GAPDH	1,01548602	0,31258589	0,38051703	tfam	1,11	1	0,65
	1,04186051	0,3583624	0,38685782		0,26	1,09	0,67
	0,86207996	0,35209387	0,32019759		0,44	0,47	0,37
	1,13792004	0,42434603	0,54650626		1,94	0,59	0,2
	0,93820794	0,47621127	0,92426646		1,54	0,15	0,12
	1,09762801	0,37944862	0,73564187		2,61	0,19	
	0,96416405	0,32374003	0,42162912				
PINK1/ GAPDH	0,60152792	1,60490133	0,6562918	NRF1	1,59	0,88	0,88
	1,02763887	1,07633808	0,69709542		0,36	1,06	1,02
	1,37083321	1,09162965	0,51413129		0,47	0,36	0,46
	1,45486241	1,94126593	0,45171922		1,2	0,5	0,17
	1,18685648	2,14289946	1,25583739		0,86	0,15	0,13
	0,81314352	1,14398937	1,02269218		3,67	0,21	
	1,01703237	1,16564801	0,87648454				

	18% Diet	1% Diet	1% Diet + Rapa
ATP nmol/g	2,766	1,278	1,388
	2,34	1,171	2,227
	1,761	1,365	1,778
	2,035	1,478	2,034
	1,888	1,715	1,884
	2,322	1,206	1,639

Supplementary Table S9 – Carbon chain metabolism

Mouse liver_ (nmol/g)	total protein (ug)	Tissue weight (mg)	Erythrose-4P	Glucose	Glucose-6P	Glycerylaldehyde-3P	Mannose-6P	Ribose-5P	a-Hydroxyglutaric acid	a-Ketoglutaric acid	Citric acid	Fumaric acid	Glycolic acid
36_BT3	235,48	28,85	0,4664	1357	20,7	6,401	5,666	60,13	36,12	11,37	118,4	520,5	30,69
37_BT4	333,60	39,31	0,8963	1064	22,58	154,1	13,86	143,7	33,03	7,619	59,56	540,8	27,03
38_BW1	306,14	41,34	0,8653	736,2	22,85	95,95	7,687	115,9	17,29	4,242	37,23	430,8	28,36
39_BW3	378,37	33,98	0,8076	869,9	20,69	98,45	4,373	123,1	31,82	8,584	62,63	569,8	32,45
40_CF1	220,58	27,95	0,9167	2233	50,25	35,76	28,93	81,05	20,85	8,254	78,2	656,2	29,19
41_BT1	253,53	30,26	1,508	1457	135,7	21,92	68,04	69,39	5,848	7,777	228,7	484,2	48,96
42_BT2	200,68	27,5	1,144	3034	54,75	6,327	40,04	54,39	6,414	5,957	135,8	517	46,53
43_BV4	303,66	31,56	1,153	878,3	97,99	21,17	35,42	59,93	5,998	5,38	184,1	465,5	50,21
44_CE1	202,86	25,02	0,6255	758,6	39,88	8,274	19,46	56,33	6,249	5,036	143	353	35,89
45_CB1	215,45	28,47	0,8621	573,6	22,01	7,572	11,87	65,98	8,976	5,49	47,25	534,5	53,27
46_BT6	287,69	28,31	0,8758	2343	41,79	13,74	8,805	138	7,162	10,61	158	1005	36,52
47_BT7	329,52	32,6	0,8427	1303	72,78	11,89	31,05	61,52	4,595	5,696	144,4	712,2	30,36
48_BT8	303,15	33,99	0,9209	4097	60,36	6,309	10,85	38,67	6,468	7,644	133,9	757,3	34,47
49_CC2	295,18	32,7	0,658	1418	32,93	8,292	16,83	60,4	4,837	5,43	54,25	288,8	30,44
50_CA2	303,30	30,83	0,7383	1396	17,96	6,649	7,415	46,61	8,242	9,462	112,1	404,5	31,4

Isocitric acid	Latic acid	Malic acid	Pyruvic acid	Succinic acid	AMP	ADP	ATP	GMP	GDP	GTP	UMP	UDP	UTP	cyclic-ADP-ribose	cyclic-AMP
3,537	5613	691,2	47,61	1,869	2,611	1,345	2,153	0,741	0,322	0,135	1,876	0,037	1,684	1,561	0,015
1,744	3310	656,2	34,48	2,53	12,07	7,305	2,63	8,008	0,717	0,079	49,679	0,228	2,253	4,427	0,0272
1,744	3176	604,1	38,2	3,231	42,43	8,595	2,528	40,148	0,648	0,088	204,067	0,672	4,254	2,626	0,0291
1,968	4780	778,2	49,64	7,987	12,585	6,071	2,606	24,263	0,463	0,111	103,083	0,381	4,411	1,961	0,0266
1,295	7882	821	55,75	3,871	32,594	8,652	3,179	22,018	0,592	0,201	85,541	0,463	4,652	2,791	0,0366
7,35	5498	652,9	41,16	3,72	19,938	7,438	4,262	10,483	0,567	0,246	51,314	0,564	7,193	2,913	0,0364
4,21	5765	800,5	55,83	6,202	8,353	5,392	1,841	9,8	0,872	0,089	48,308	0,482	5,734	2,028	0,0339
8,47	1979	1003	34,88	3,173	6,398	6,544	1,677	3,471	0,818	0,088	33,107	0,355	4,136	2,003	0,0255
5,779	3180	536,5	37,87	2,755	8,248	5,033	2,026	6,814	0,645	0,123	61,283	0,278	3,652	1,858	0,0302
3,986	1390	581,7	34,2	2,502	3,779	3,453	1,735	1,78	0,523	0,142	10,207	0,138	2,559	1,476	0,0259
3,762	5389	1487	84,25	27,8	4,229	4,345	1,994	3,243	0,486	0,183	38,337	0,262	3,139	1,961	0,0272
5,333	4666	955,4	100,1	4,667	6,679	4,156	3,703	1,921	0,404	0,318	7,596	0,13	3,941	2,57	0,0253
2,641	7090	1453	47,37	13,19	4,324	4,704	4,838	1,314	0,297	0,243	9,162	0,105	5,583	1,538	0,0154
1,968	3729	437,8	39,99	3,012	8,44	5,038	2,819	2,999	0,575	0,265	19,641	0,26	3,523	2,125	0,0248
3,089	4635	507,7	39,68	4,513	6,993	5,492	1,706	6,657	0,717	0,053	42,675	0,175	2,897	2,358	0,0228

6-P-Gluconate	Acetylglucosamine-1P	Acetyl-Phosphate	ADP-Glucose	2,3-bisP-Glycerate	DHAP	total of Fructose-bisP/Glucose-1,6-bisP	Fructose-6P	Glucosamine-6P	Glycerate-2- or 3-P	Glycerol-3-P	PEP	Phosphocreatine	Ribulose-1,5-bisP
8,2431	4,9889	15,838	0,01929	0,1301	1,466	0,538	29,109	1,314	13,37	32,286	6,486	0,033	0,255
14,2842	7,727	27,382	0,2507	0,2857	1,255	0,262	22,222	1,357	2,179	6,462	1,63	Not quan	0,652
7,2694	5,0571	38,781	0,41968	0,1428	18,833	0,177	36,619	0,947	1,961	6,094	1,164	Not quan	0,454
8,4632	7,4168	23,202	0,28529	0,2265	7,087	0,313	29,3	0,831	2,441	7,21	1,321	Not quan	0,635
7,0512	5,7329	28,277	0,30213	0,1662	10,975	0,219	40,288	1,52	2,141	6,552	1,437	Not quan	0,516
11,4021	7,6582	30,767	0,57683	0,0858	8,91	0,247	48,674	0,875	1,892	3,779	1,472	Not quan	0,517
8,2355	6,9241	60,626	0,26254	0,0856	5,717	1,543	101,051	0,886	1,504	7,14	1,508	0,007	0,318
12,8332	7,0886	60,048	0,22759	0,126	2,215	0,781	42,49	0,798	1,626	7,866	1,461	0,057	0,498
5,9592	7,4531	46,403	0,24026	0,0475	6,038	1,55	60,564	0,91	1,435	10,022	1,349	Not quan	0,435
5,5425	9,8974	38,114	0,06619	0,1438	1,931	0,664	24,822	0,713	1,481	5,343	1,33	0,039	0,378
5,794	8,5021	42,623	0,30117	0,0778	1,063	0,368	17,673	1,041	0,882	4,465	1,088	0,012	0,411
9,939	6,2831	34,167	0,06987	0,093	5,131	1,711	35,637	1,596	2,387	40,875	1,205	0,01	0,415
10,1074	9,2099	43,668	0,02376	0,0238	3,298	0,828	59,432	0,763	9,481	5,878	4,54	0,028	0,239
10,4223	3,4361	36,582	0,15676	0,1277	4,242	1,225	40,916	1,452	1,462	14,998	1,474	Not quan	0,46
8,1977	5,1579	44,37	0,32001	0,1396	2,204	0,427	22,259	0,606	1,347	4,119	1,25	0,027	0,455

Ribulose-5P	Sedoheptulose-7P	UDP-Glucose	Acetoacetyl-CoA	Acetyl-CoA	Hs-CoA	Malonyl-CoA	Succinyl-CoA	NAD+	NADH	NADP+	NADPH
36,197	31,325	8,604	Not quantifiable	0,00375	0,06227	0,01185	0,55515	0,92326	2,51267	0,48265	0,02126
97,616	110,969	33,466	Not quantifiable	0,00663	0,05849	0,00792	1,09583	0,63467	2,18771	0,54388	0,02391
484,816	4,504	87,607	Not quantifiable	0,00322	0,01116	0,0048	2,66523	0,46992	1,99997	0,25066	0,01476
239,824	3,224	79,682	Not quantifiable	0,00503	0,06131	0,01087	1,05679	0,48186	3,18168	0,49336	0,02594
208,235	1,778	159,833	Not quantifiable	0,00568	0,37892	0,01046	2,5881	0,62013	1,62486	0,19507	0,02441
254,773	5,374	51,647	Not quantifiable	0,0041	0,16647	0,00999	1,14092	0,39259	1,26678	0,54887	0,0132
131,632	176,976	68,269	Not quantifiable	0,00325	0,31087	0,00568	0,68944	0,29694	0,69722	0,53456	0,0173
112,122	376,613	23,32	Not quantifiable	0,00336	0,14463	0,00595	0,77381	0,38943	1,24553	0,46662	0,01201
119,325	126,67	49,117	Not quantifiable	0,00246	0,09063	0,00777	0,75031	0,20781	1,55375	0,58034	0,00902
83,181	255,391	12,989	Not quantifiable	0,00262	0,14433	0,00886	0,95269	0,34941	1,86591	0,30438	0,0132
100,254	289,964	59,405	Not quantifiable	0,00349	0,4451	0,00792	3,06818	0,44762	0,71057	0,70916	0,01516
71,547	38,288	50,178	Not quantifiable	0,00428	0,12167	0,00552	0,45305	0,31296	0,87424	0,36892	0,00792
108,531	115,63	15,307	Not quantifiable	0,00329	0,55143	0,01069	1,83902	0,3991	0,64575	0,71119	0,01835
95,504	124,88	63,02	Not quantifiable	0,00438	0,20575	0,01858	0,8382	0,3807	1,67268	0,75062	0,01073
108,006	309,875	43,525	Not quantifiable	0,00217	0,32751	0,00247	0,141	0,3637	0,53845	0,33037	0,01505
